# Ethnobotanical survey of medicinal dietary plants used by the Naxi People in Lijiang Area, Northwest Yunnan, China

**DOI:** 10.1186/s13002-015-0030-6

**Published:** 2015-05-12

**Authors:** Lingling Zhang, Yu Zhang, Shengji Pei, Yanfei Geng, Chen Wang, Wang Yuhua

**Affiliations:** Kunming Institute of Botany, Chinese Academy of Sciences, Kunming, 650201 China; South China Botanical Garden, Chinese Academy of Sciences, Guangzhou, 510650 China; University of Chinese Academy of Sciences, Beijing, 100049 China

**Keywords:** Medicinal dietary plant, Traditional Chinese medicine, Ethnobotany, Naxi, Lijiang

## Abstract

**Background:**

Food and herbal medicinal therapy is an important aspect of Chinese traditional culture and traditional Chinese medicine. The Naxi are indigenous residents of the Ancient Tea Horse Road, and the medicine of the Naxi integrates traditional Chinese, Tibetan, and Shamanic medicinal systems, however, little is known about the medicinal dietary plants used by the Naxi people, or their ethnobotanical knowledge. This is the first study to document the plant species used as medicinal dietary plants by the Naxi of the Lijiang area.

**Methods:**

Ethnobotancial surveys were conducted with 89 informants (35 key informants) from 2012 to 2013. Three different Naxi villages were selected as the study sites. Literature research, participatory investigation, key informant interviews, and group discussions were conducted to document medicinal dietary plants and the parts used, habitat, preparation methods, and function of these plants. The fidelity level (FL) was used to determine the acceptance of these medicinal dietary plants. Voucher specimens were collected for taxonomic identification.

**Results:**

Surveys at the study sites found that 41 ethnotaxa corresponded to 55 botanical taxa (species, varieties, or subspecies) belonging to 24 families and 41 genera. Overall, 60 % of documented plants belonged to seven botanical families. The most common families were Compositae (16.4 %) and Rosaceae (10.9 %). Roots (34.1 %) were the most common part used. Wild-gathered (68.3 %), semi-domesticated (17.1 %), and cultivated (14.6 %) were the most common habitats of medicinal dietary plants. Stewing plants with meat was the most common preparation and consumption method. The plants were used to treat 21 major health conditions; alleviating fatigue (42.8 %) was the most common. The maximum FL of 100 was found for 68.3 % of the medicinal dietary plants

**Conclusions:**

The medicinal dietary plants used by the Naxi people are diverse and are used to treat a wide spectrum of body disorders. Further studies focusing on safety, detoxification, and nutritional value of the plants should be conducted to allow them to be used to improve health and prevent diseases in modern society.

## Background

There is much overlap between medicine and food [1–5], and dietary products can simultaneously be a food and a medicine [2–4, 6–11]. In fact, many plants in local food cultures are inseparable from traditional therapeutic systems [8, 12–15]. China has a long cultural history of homologous medicine and food, and the thought of “food as medicine” has existed in China since ancient times [16, 17]. Therefore, food therapy is an important characteristic of Chinese culture and traditional Chinese medicine (TCM). The concept of food therapy was proposed 2000–3000 years ago in the *Inner Canon of Huangdi* [18]. In the Tang Dynasty, food was used to treat diseases and the famous medical expert Sun Simiao proposed that “the healer must know the causes of the disease and the disorder of the body, then first treat with food, if it does not work, then medicine could be adopted” [19]. Food therapy as a part of TCM is based on the Chinese philosophy of Yin -Yang and the Five Elements (metal, wood, water, fire and earth) [20]. Food therapy uses holistic principles as its base, and highly emphasizes harmony within the universe [20, 21], while preventing disease by enhancing the condition of the body.

A medicinal diet includes using medicines in the diet or functional foods used as medicines, based on the current health-status of an individual, as well as the overall epidemiological situation of a population [19, 21]. Because the population is aging and the number of people who are sub-healthy and living with chronic diseases is increasing, the Chinese medicinal diet has received considerable attention. In the United States, Europe, and Australia, the “non-nutritive” health roles of diet are receiving increasing attention within the areas of functional foods, nutraceuticals, and phytonutrients [15, 22–26]. Since 1985, more than ten food therapy books per year have been published in China [27]. However, study of the medicinal diets used by indigenous communities in China has been mostly neglected.

TCM has spread to Lijiang and was adopted by the Naxi people [28]. The population of the Naxi people in China is about 324,679 and mainly they inhabits the Yulong Naxi nationality Autonomous County in the Lijiang area [29]. The Naxi are a Burmo-Naxi-Lolo sociolinguistic sub-group of the Tibeto-Burman group within the Sino-Tibetan family [30]. The Naxi population was formed during the southward migration of the ancient Qiang people during the Qin Dynasty (221–206 BC), who had originally inhabited the Hehuang area of Northwest China [30]. The Naxi are indigenous residents of the Ancient Tea Horse Road, a trade link documented since the Tang dynasty (618–907 CE) which lasted until the 1960s, and stretched across Yunnan, Sichuan and Tibetan provinces [31]. The road promoted exchanges in culture, religion and ethnic migration, resembling the Silk Road [32]. Given this history, the medicine of the Naxi integrates traditional Chinese, Tibetan, and Shamanic medicinal systems [28]. Prior research indicates that the Naxi culture promotes diet therapy, and documentation of many of their traditional medicines and diet remedies exists through the world’s only remaining pictographic writing system [33]. Despite the renewed interest in medicinal diets by scientists, consumers, and industry, not much is known about the medicinal dietary plants used by the Naxi, or their associated ethnobotanical knowledge. This is the first study to focus on the medicinal dietary plants of the Naxi People and their associated knowledge. The plant materials, parts used, habitats, and medicinal dietary uses are recorded and the fidelity of the medicinal dietary plants is assessed.

## Methods

### Study site

**Fig. 1:**
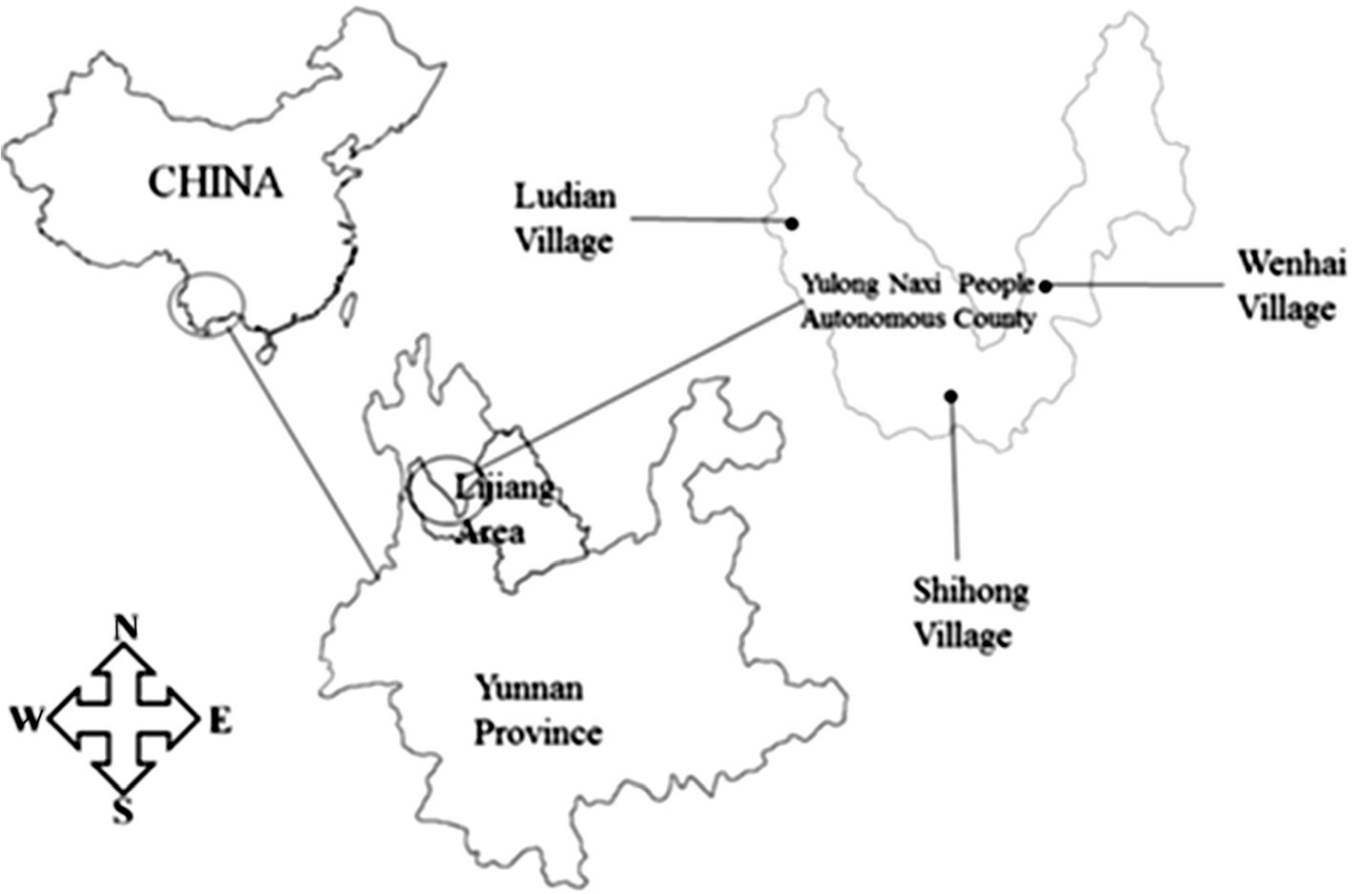
Location map of study sites

The study was conducted in the Yulong Naxi Nationality Autonomous County in Lijiang Area in Northwest Yunnan Province, China. Northwest Yunnan is located in the Three Parallel Rivers region, and Lijiang is listed as a World Natural and Cultural Heritage Site by UNESCO. The region’s exceptional altitudinal range, topography, and climatic variability have fostered centers of plant species endemism [34–36]. Northwest Yunnan harbors over 3500 endemic plant species, many of which are used by local communities including the Naxi [34, 35, 37]. Three typical Naxi villages (Wenhai, Ludian, and Shihong) were selected as study sites. These villages represent particular landforms in the Northwest Yunnan plateau, and have different positions in the Naxi culture (Fig. 1).

Wenhai village is a typical wet plateau basin located directly on the western foot of the first peak of the Yulong Snow Mountain. The location is 26°59′16_._37″E, 100°10′6.23″N and the altitude is between 3109 and 3380 m. Weihai village is an ancient village on the Ancient Tea Horse Road leading to Tibet [32]. Ludian village is a typical dry plateau basin located at the center of the Northwest Yunnan plateau. The location is 103°19'12"E, 27°7′48″N and the altitude is between 2400 and 2800 m. Ludian village is the last stop of the “Soul Sending Way”, which is the transfer road of the Naxi [30]. Shihong village is a typical mountain village located on the southwestern most peak, “Ninety-nine Longtan”, of the Laojun Mountain. The location is 26°41′24″E, 99°53′49″N, and the altitude is between 3500 and 3600 m. The village was the first settlement in the surrounding area [38], and both the traditional culture and natural environment are well preserved.

### Data collection

The data were collected from 2012 to 2013, and at least one half of one month of the fieldwork was conducted in each season every year. The methods we adopted included literature research, participatory investigation, key informant interviews, and group discussions [39]. Eighty-nine informants (49 men and 40 women) were interviewed. Informant ages ranged from 19 to 85 years old, and those aged above 60 were key informants. The interviews were carried out in standard Mandarin Chinese given that most of the population is bilingual and has attended a Chinese language school. We recorded the local Chinese language names as well as local Naxi names.

Our study began with a literature search, which not only helped identify proper study sites, but also helped us understand flora of Northwest Yunnan and collect the ethnobotanical data of the Naxi. The first step of the fieldwork was participatory investigation, and the main task was to search for medicinal dietary plants with the key informants to perform a quick inventory, collect voucher specimens, record habitats, and take photos. The preparation and consumption procedures were witnessed and recorded in the homes of the villagers. After that, key informant interviews were performed. The plant specimens collected in the participatory investigation were used for reference during the key informant interviews. In the key informant interviews, detailed information about each plant, such as the local Chinese and Naxi names, habitat, edible parts, preparation, consumption, and medicinal dietary function, were documented. During group discussions, the information garnered in the first two stages was discussed by a group of informants to ensure accuracy.

### Fidelity level

Fidelity level (FL) was used to assess the acceptance of each medicinal dietary plant at the ethnotaxic level. The formula used was FL = Np/N × 100 [40–42], where Np is the number of informants stating the use of the plant as a medicinal dietary plant and N is the total number of informants citing the plant as edible or for medicinal use. As medicinal dietary plants are edible plants can be used as medicine (for example *Allium tuberosum,* a common vegetable, can be used to calm nerves) or medicinal plants can be consumed with food (for example *Aconitum stapfianum* is consumed by stewing with meat in winter for its anti-rheumatic benefits), we define N as the total number of informants citing the plant for edible or medicinal use. The values of FL range from 0 to 100, and increasing values of FL for a plant indicate its greater acceptance as a medicinal dietary plant.

This study was carried out following the code of ethics of the American Anthropological Association [43, 44] and the International Society of Ethnobiology Code of Ethics [40, 45]. Prior oral informed consent was acquired. Specimen identification was completed with the help of the experts at the Kunming Institute of Botany, and these specimens will be stored in the Herbarium of the Kunming Institute of Botany at the Chinese Academy of Sciences after our further study.

## Results

**Fig. 2:**
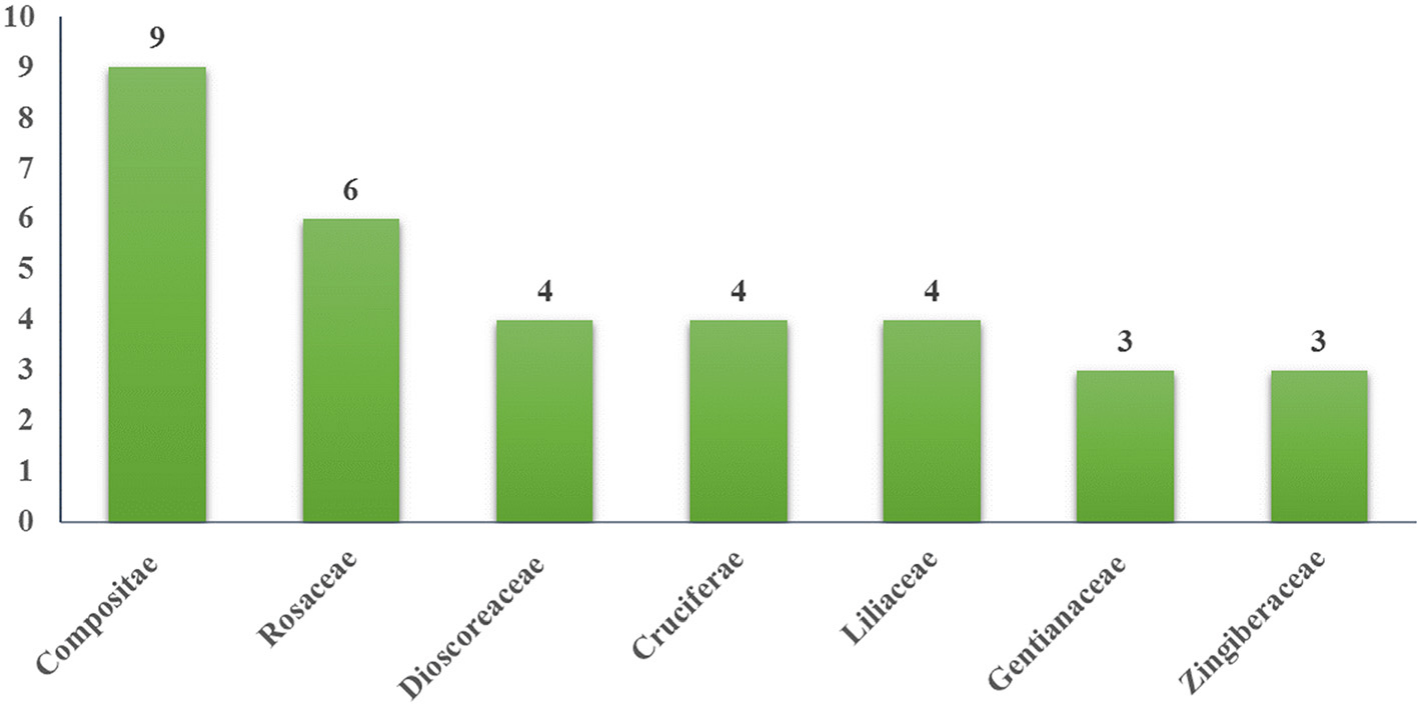
Most quoted plant families by number of species included

Ethnobotanical surveys at the study sites found that 41 ethnotaxa of medicinal dietary plants correspond to 55 botanical taxa (species, varieties, or subspecies) belonging to 24 families and 41 genera. At the family level, we found that 60 % of documented plants belonged to seven botanical families, and the most represented families were Asteraceae (16.4 %), Rosaceae (10.9 %), Dioscoreaceae (7.4 %), Cruciferae (7.4 %), and Liliaceae (7.4 %) (Fig. 2). These families were similar to other edible plants recorded in the same site, of which Rosaceae (19.8 % species) and Liliaceae (10.3 % species) were the most common. However, the similarity with the medicinal plants of the Naxi is apparently larger, of which Asteraceae (13.1 %) and Labiatae (5.8 %) were the most common family [33]. At the species level, those medicinal dietary plants accounted for 39 % of the total number of edible plant species (146) recorded at the study sites. However, these plants had less overlap with recorded Naxi medicine plants [30]. This indicates that the selection of medicinal dietary plants overlaps with the edible and medicinal plants in the area. However, previous studies may have treated some plants as edible or medicinal only, and the medicinal dietary functions were ignored. Table 1 lists the ethnobotanical information for each plant, including the scientific name, local name, family name, habitat, usage, preparation, and plant part used.

**Table 1:** Inventory of medicinal dietary plants used by the Naxi in Lijiang area, Northwest Yunnan, China (Ranked alphabetically, Ethnotaxically organized)

Local name	Family	Scientific name of original plant	Medicinal dietary function	Diet type	Preparation	Part used	Habitat	Voucher specimen code
Zhuzishen	Campanulaceae	*Codonopsis convolvulacea* subsp. *forrestii* (Diels) D. Y. Hong ex L. M. Ma	Treat eye disease	Medicinal diet	Stewed with meat; steamed with egg	Root	Wild-gathered	ZLL-0005
Shanyao	Dioscoreaceae	*Dioscorea deltoidea* Wall. ex Griseb.; *Dioscorea delavayi* Franch.; *Dioscorea hemsleyi* Prain ex Burkill; Dioscorea sp.	Alleviate fatigue, Tonifying	Vegetable, Medicinal diet	Stewed with meat	Root	Semi-domesticated	ZY-0021, ZY-0022, ZY-0023, ZY-0024
Yekuai	Malvaceae	*Malva verticillata* L.	Postpartum blood stasis	Medicinal diet	Soup made with egg	Root	Wild-gathered	ZLL-0001
Xuelianhua	Compositae	*Saussurea leucoma* Diels	Alleviate fatigue, Tonifying	Medicinal diet	Soaked in boiling water	Whole plant	Wild-gathered	ZLL-0010
Xuecha	Icmadophilaceae	*Thamnolia vermicularis* (Sw.) Ach.ex Schae; *Thamnolia subuliformis* (Ehrh.) W. L. Culb	Treat cold, Clean away heat, detoxification	Generation tea	Soaked in boiling water	Whole plant	Wild-gathered	ZLL-0079, ZLL-0080
Xiaohongshen	Rubiaceae	*Galium elegans* Wall. ex Roxb.	Alleviate backache,bruises	Medicinal diet	Stewed with meat	Root	Wild-gathered	ZY-0035
Xiangyashen	Zingiberaceae	*Roscoea tibetica* Batalin; *Roscoea yunnanensis* Loes.; *Roscoea cautleoides* Gagnep.	Kidney deficiency	Medicinal diet	Stewed with meat	Root	Wild-gathered	ZY-0121, ZY-0122, ZY-0123
Xiangru	Labiatae	*Origanum vulgare* L.	Treat cold	Spices	Boil water	Aerial part	Wild-gathered	GYF-0061
Wuweizi	Magnoliaceae	*Schisandra chinensis* (Turcz.) Baill.	Alleviate fatigue,treat insomnia	Fruit, medicinal diet	Infused with alcohol	Fruit	Wild-gathered	ZLL-0027
Tiaoshen	Compositae	*Stebbinsia umbrella* (Franch.) Lipsch.	Alleviate fatigue, Tonifying	Medicinal diet	Stewed with meat	Root	Wild-gathered	ZLL-0107
Songhuafen	Pinaceae	*Pinus armandii* Franch.	Moistening lung, Anti-tussive	Medicinal diet	Mixed with honey or brown sugar	Pollen	Wild-gathered	ZLL-0060
Songzi	Pinaceae	*Pinus yunnanensis* Franch.	Moistening lung, Anti-tussive	Nut	Dried	Kernel	Wild-gathered	ZY-0074
Songluo	Parmeliaceae	*Usnea longissina* Ach.	Moistening lung, Anti-tussive	Generation tea	Soaked in boiling water	Whole plant	Wild-gathered	GYF-0082
Songjisheng	Loranthaceae	*Arceuthobium pini* Hawksworth et Wiens	Treat insomnia	Generation tea	Soaked in boiling water	Leaf	Wild-gathered	ZLL-0105
Shanzha	Rosaceae	*Crataegus scabrifolia* (Franch.) Rehd.; *Crataegus chungtienensis* W. W. Smith	Appetizing,diuresis	Fruit, Candied fruit	Soaked in boiling water,mixed with honey	Fruit	Semi-domesticated	ZLL-0016, ZLL-0017
Shanjinzi	Rosaceae	*Malus rockii* Rehd.	Appetizing,diuresis	Fruit, Candied fruit	Soaked in boiling water,mixed with honey	Fruit	Semi-domesticated	ZLL-0132
Qinciguo	Rosaceae	*Prinsepia utilis* Royle	Postpartum weakness, Tonifying,	Oil	Extracted oil	Kernel	Semi-domesticated	ZLL-0075
Pugong Ying	Compositae	*Taraxacum mongolicum* Hand.-Mazz.	Clean away heat, detoxification	Vegetable	Made into soup	Aerial part	Wild-gathered	ZY-0002
Niubang	Compositae	*Arctium lappa* L.	Alleviate fatigue, Tonifying	Medicinal diet	Stewed with meat	Root	Wild-gathered	ZY-0007
Mugua	Rosaceae	*Chaenomeles speciosa* (Sweet) Nakai	Anti–rheumatism	Fruit, seasoning	Stewed with fish	Fruit	Wild-gathered	ZLL-0013
Muer	Auriculariaceae	*Auricularia auricula* (L. ex Hook.) Underw	Gut purge	Vegetable	Cold dish	Whole plant	Wild-gathered	ZLL-0028
Mianshen	Labiatae	*Eriophyton wallichii* Benth.	Alleviate fatigue, Tonifying	Medicinal diet	Stewed with meat	Root	Wild-gathered	ZLL-0065
Manjing	Cruciferae	*Brassica rapa* L.	Appetizing	Grain, vegetable	Raw, boiled	Root	Domesticated	ZLL-0045
Luobo	Cruciferae	*Raphanus sativus* L.	Appetizing	Vegetable	Raw, boiled	Root	Domesticated	ZLL-0069
Longdancao	Gentianaceae	*Gentiana rigescens* Franch. ex Hemsl.; *Gentiana szechenyii* Kanitz; *Gentiana cephalantha* Franch. ex Hemsl.	Kidney deficiency	Ferment	Boiled in water	Aerial part	Wild-gathered	ZY-0094, ZY-0095, ZY-0096
Juehuashen	Compositae	*Hippolytia delavayi* (Franch. ex W. W. Smith) Shih	Alleviate fatigue, Tonifying, moistening lung, Anti-tussive	Medicinal diet	Stewed with meat	Root	Wild-gathered	ZY-0039
Jiucai	Liliaceae	*Allium tuberosum* Rottl. ex Spreng.	Calm nerves	Vegetable	Cooked with goat liver	Leaf	Domesticated	ZLL-0032
Jicai	Cruciferae	C*apsella bursa-pastoris* (Linn.) Medik.	Clean away heat, detoxification	Medicinal diet	Stewed with meat	Root	Wild-gathered	ZY-0054
Daji	Compositae	*Cirsium griseum* Levl.; *Cirsium lidjiangense* Petr. ex Hand.-Mazz.; *Cirsium chlorolepis* Petr. ex Hand.-Mazz.; *Cirsium eriophoroides* (Hook.f.) Petrak	Tonifying	Medicinal diet	Stewed with meat	Root	Wild-gathered	ZLL-0041, ZLL-0042, ZLL-0043, ZLL-0044
Huixiang	Umbelliferae	*Foeniculum vulgare* Mill.	Alleviate fatigue,alleviate backache	Vegetable, seasoning	Steamed with egg	Tender stem	Cultivated	GYF-0108
Huitiaocai	Chenopodiaceae	*Chenopodium album* L.	Treat constipation	Vegetable	Made into soup	Tender stem	Wild-gathered	ZLL-0128
Huangjing	Liliaceae	*Polygonatum cirrhifolium* (Wall.) Royle	Anti–rheumatism, promoting lactation,	Medicinal diet	Brewed	Root	Wild-gathered	ZLL-0078
Huajiao	Rutaceae	*Zanthoxylum bungeanum* Maxim.	Anti-rheumatism, treat belly ache	Seasoning	Soaked in boiling water	Fruit	Semi-domesticated	ZLL-0056
Heicaowu	Ranunculaceae	*Aconitum stapfianum* Hand.-Mazz.	Anti-rheumatism, treat stomachache, belly ache	Medicinal diet	Stewed with meat	Root	Wild-gathered	ZLL-0067
Elancai	Cruciferae	*Thlaspi arvense* L.	Diuresis	Vegetable	Made into soup	Aerial part	Wild-gathered	ZLL-0029
Dujuanhua	Ericaceae	*Rhododendron yunnanense* Franch; *Rhododendron hippophaeoides* Balf. f. et W. W. Smith	Gut purge	Snacks, vegetable	Cold dish	Petal	Wild-gathered	ZY-0037, ZY-0038
Chuanxiong	Umbelliferae	*Ligusticum chuanxiong* Hort.	Alleviate backache	Vegetable	Steamed with egg	Tender stem	Cultivated	ZLL-0104
Chonglian	Rosaceae	*Sanguisorba filiformis* (Hook. f.) Hand.-Mazz.	Kidney deficiency	Medicinal diet	Stewed with meat	Root	Wild-gathered	ZLL-0097
Chongcao	Clavicipitaceae	*Cordyceps sinensis* (Berk.) Sacc.	Treat eye disease	Medicinal diet	Stewed with meat, steamed with egg,infused with alcohol	Whole plant	Wild-gathered	ZLL-0077
Cheqiancao	Plantaginaceae	*Plantago depressa* Willd.	Alleviate fatigue,diuresis	Vegetable	Stewed with meat	Root	Wild-gathered	ZLL-0116
Baihe	Liliaceae	*Lilium lankongense* Franch.; *Lilium lancifolium* Thunb.	Moistening lung, Anti-tussive	Vegetable	Stewed with meat	Bulb	Wild-gathered	GYF-0102, GYF-0103

### Used parts

**Fig. 3:**
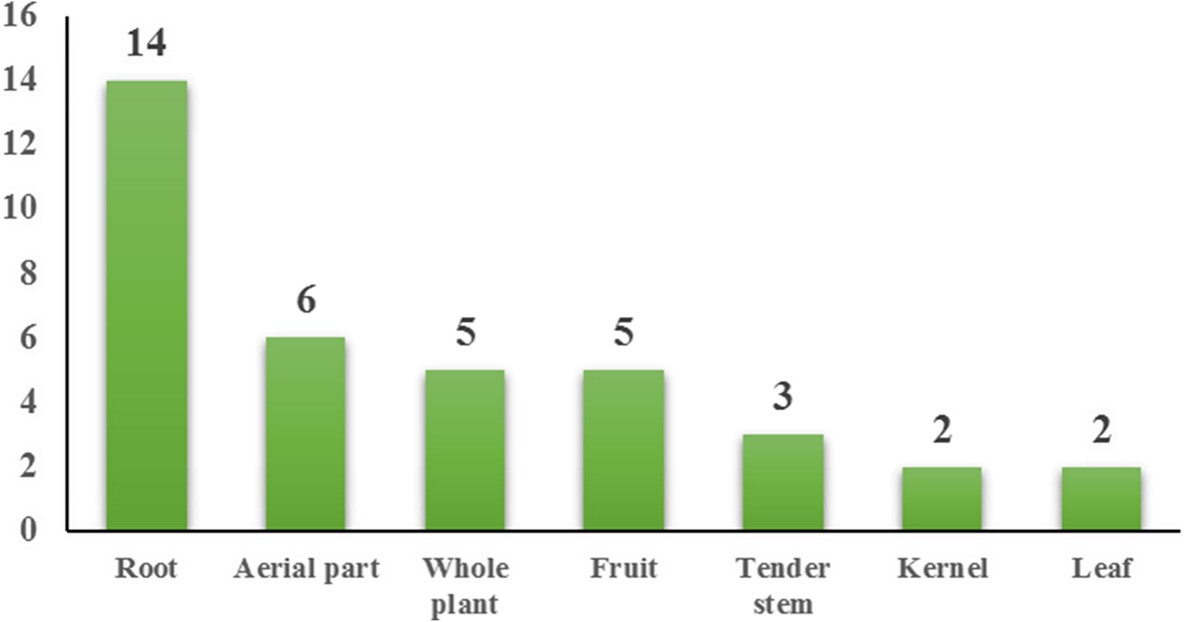
Plants parts used in the medicinal diet

A wide range of plant parts including root, aerial plant, whole plant, fruit, tender stem, kernel, leaf, bulb, pollen, petal, and rhizome or flower from herbaceous (75.6 %) or woody plants (24.4 %) are used. The root was the most commonly used plant part (34.1 %); the most common seven plant parts are shown in Fig. 3. The remaining less commonly used parts were the bulb, pollen, petal, and rhizome. These results share little similarity to other edible plants, of which the fruit (44.8 %) and leaf (30.5 %) are the most commonly used parts. However, the used parts were similar to those on the Naxi medicinal plant list, with root as the most commonly used part (43.8 %) [33]. The informants stated that roots contain more substances for good health. Similar thinking is found in TCM, with the use of *Panax ginseng* as a prominent example.

### Preparation

Mixing plants with other food items was the most common preparation and consumption method. The majority of inventoried medicinal dietary plants were stewed with meat (34.1 %), followed by use in soup, soaking in boiled water, and steaming with eggs. There were a few other preparations that involved infusion with alcohol, or mixing with honey or brown sugar. The Naxi believe that the meat of an animal has feelings, so it is better to enhance the blood and energy of the human body and prolong life. This concept is also recorded in the *Southern Yunnan Materia Medica* [46]. Nearly all remedies include only one medicinal dietary plant, and are easy to prepare.

### Habitat

**Fig. 4:**
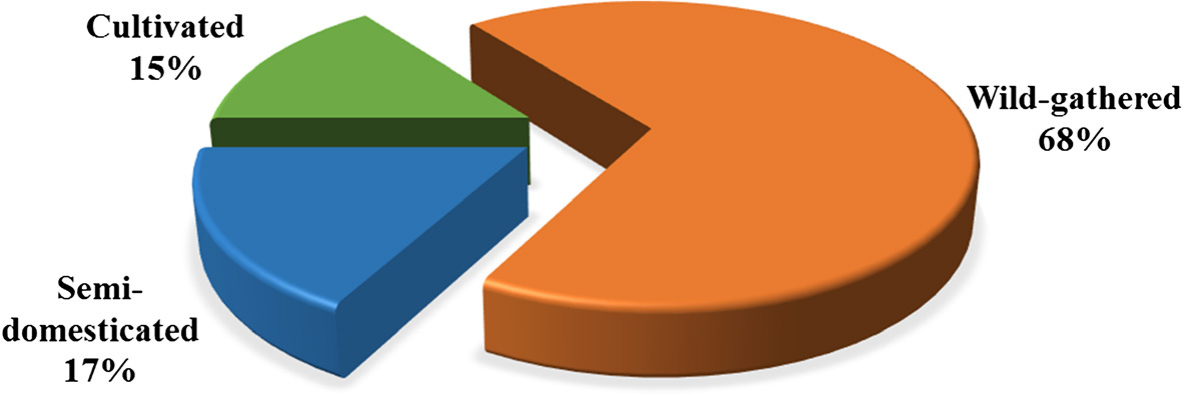
The habitat of medicinal dietary plants

Wild-gathered (68.3 %), semi-domesticated (17.1 %), and cultivated (14.6 %) were the most common habitats of medicinal dietary plants (Fig. 4). The majority of the medicinal dietary plants of the Naxi were gathered from the wild. The Naxi prefer medicinal dietary plants that grow on high mountains, as they believe that these plants are more effective and impart longevity. The Naxi in Wenhai village liked to collect medicinal dietary plants from the Yulong Snow Mountain, and in Shihong village the Naxi collected plants from the Laojun Mountain. The most representative plants were *Cordyceps sinensis* and *Saussurea leucoma*. Semi-domesticated plants referred to those plants cultivated in home gardens. The Naxi prefer to grow medicinal plants which collected from the mountains in their home gardens [34], with a part for medicinal dietary use.

### Medicinal dietary function

**Fig. 5:**
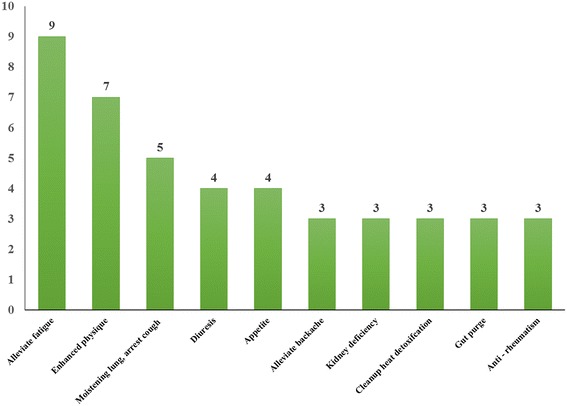
Medicinal diet function category-number of plants used

There are 21 major health conditions that can be treated by the collected medicinal dietary plants. The most common condition that can be treated is alleviating fatigue (42.8 %), followed by tonifying, moistening the lung, and as use as an antitussive (Fig. 5). In addition to the ten types of medical functions shown in Fig. 5, there were ten other functions including treating eye diseases, insomnia, cold, stomachache, abdominal pain, bruises, constipation, postpartum blood stasis, postpartum weakness, calming of the nerves, and promotion of lactation.

### Fidelity level

Sixty-eight percent of medicinal dietary plants used by the Naxi people had a maximum FL value of 100 and 83 % of the edible dietary plants had a FL value of over 50. This indicates the general acceptance of inventoried plants as medicinal dietary plants within the Naxi community. Special emphasis was given to some important plants that have maximum FL value. These plants included *Polygonatum cirrhifolium*, whose tuber is commonly used for promoting lactation, and is a widely used vegetable [47]. Others were *Pinus armandii*, *Cirsium griseum*, *Cirsium lidjiangense*, *Cirsium chlorolepis*, *Cirsium eriophoroides*, *Ligusticum chuanxiong, Zanthoxylum bungeanum,* and *Aconitum stapfianum,* which are wildly enjoyed by Naxi people for their good effect. Detailed use and the FL values of medicinal dietary plants are shown in Tables 1 and 2.

**Table 2:** Fidelity level of medicinal dietary plants of the Naxi

Local name	Medicinal dietary function	FL
Songhuafeng	Moistening the lung, Antitussive	100
Chuanxiong	Alleviates backache	100
Heicaowu	Anti-rheumatism, Treats stomachache, belly pain	100
Huajiao	Anti-rheumatism, Treats stomachache, belly pain	100
Huangjing	Anti-rheumatism, Promoting lactation	100
Chongcao	Treats eye disease	100
Ji	Tonifying	100
Songzi	Moistening the lung, Antitussive	100
Xuelianhua	Alleviates fatigue, Tonifying	100
Muer	Gut purge	100
Wuweizhi	Alleviates fatigue, Treats insomnia	100
Huixiang	Alleviates fatigue, Alleviates backache	100
Baihe	Moistening the lung, Antitussive	100
Shanjingzi	Appetizing, Diuresis	100
Shanzha	Appetizing, Diuresis	100
Manjing	Appetizing	100
Luobo	Appetizing	100
Xuecha	Treats colds, cleanup heat and detoxification	100
Mugua	Anti-rheumatism	100
Qingciguo	Treats postpartum weakness, Tonifying	100
Yeshanyao	Alleviates fatigue, Tonifying	100
Niubang	Alleviates fatigue, Tonifying	100
Xiaohongshen	Alleviates backache, Treat bruises	100
Xiangyashen	Tonifying kidney	100
zhuzishen	Treats eye disease	100
Tiaoshen	Treats insomnia	100
Jiuhuashen	Alleviates fatigue, Tonifying, Moistening the lung, Antitussive	100
Mianshen	Alleviates fatigue, Tonifying	100
Xiangru	Treats colds	80
Huitiaocai	Treats constipation	80
Cheqiancao	Alleviates fatigue, Diuresis	80
Jiucai	Calms nerves	60
Songjishen	Treats insomnia	60
Yekuai	Treats postpartum congestion	50
Pugongying	Cleanup heat and detoxification	40
Longdancao	Tonifying kidney	40
Jicai	Cleanup heat and detoxification	30
Chonglian	Tonifying kidney	30
Elancai	Diuresis	30
Songluo	Moistening the lung, Antitussive	30
Dujuanhua	Gut purge	20

## Discussion

A large number of studies focusing on traditional medical diets have been conducted in China. Of these, two comparable ethnobotanical studies of medicinal dietary plants were conducted by Zhang [48] in Taibai Mountain, and by Gu [49] concerning Dai people in Xishuangbanna. Zhang’s study recorded the use of 183 species of medicinal dietary plants belonging to 61 families and 119 genera. The most represented families were Rosaceae, Liliaceae and Compositae, and the majority were in herb form (58.5 %). In Zhang's study the whole herb was used 36.6 % of the time, and the main preparation style was cold-dressed and eaten with noodles. The main medicinal functions were relieving fever and eliminating toxins. Gu documented 135 species of medicinal dietary plants, most of which were herbaceous plants, belonging to 49 families and 104 genera. Most plants were used to treat cold, indigestion, diarrhea and injuries. Gu conducted a biochemical investigation into two traditionally important medicinal dietary plants, *Gmelina arborea* and *Strobilanthes cusia*. The book *Materia Medica of Yunnan* records 73 prescription herbs and outlines the character of the Yunnan medicinal diet [46]. The results of our study share many common features with the plants used in *Materia Medica of Yunnan*, for example, nearly all prescribed medicinal diets include only one medicinal dietary plant, meat is used to enhance the beneficial effects of the plant, and liquor, honey and sugar are used to prepare the plant for consumption.

The richness of plant diversity in any area is evaluated not only by the number of species occurring there, but also by the intensity of associations and the dependence of the indigenous communities on those plants [15]. The medicinal dietary plants of the Naxi people are diverse. Villagers are knowledgeable about the use of various medicinal dietary plants to improve health, and to prevent and treat diseases. Roots are collected and used for the whole year, and green plant items are stored to prolong use through winter. The life of the Naxi people is closely related with medical dietary plants, and their medicinal diets are indispensable to the health of their communities. The changing relationship between humans and the environment has affected the medicinal diets and associated knowledge of the Naxi people. The selection of the medicinal diet overlaps with the edible and medicinal plants in the area. However, most studies have examined edible and medicinal plants separately, while the medicinal dietary functions are ignored.

Although most of the medicinal dietary plants had a maximum fidelity score and were widely used, the safety must be carefully checked. Thirty-nine percent of the medicinal diet plants are not commonly consumed food items (Table 1), although almost all of them are traditional herbs and the medicinal aspects are known. For example, *Aconitum* in its raw state is a highly toxic plant because of the compound aconitine [50]. There have been many reports of *Aconitum* poisoning and death in the Yunnan province [51], but it is still widely eaten by the Naxi. As in Qinling Mountain, the preparations of the *Aconitum* are special [50], and the process is strictly controlled to protect against toxic effects. Scientific evidence on the safety and detoxification of the medicinal dietary plants of the Naxi people must be established before widespread use.

## Conclusion

The medicinal dietary plants used by the Naxi people are diverse. The lives of the Naxi people are closely related with the use of medicinal dietary plants and their associated knowledge of these plants is extensive. These plants are easy to collect and prepare, and are widely used when needed by the Naxi people. The main theory behind the traditional medicinal diet of the Naxi people is to prevent disease by strengthening the body.

A wide spectrum of disorders can be treated by medicinal diets. Most plants have a high fidelity level and are widely used. However, the safety of some medicinal dietary plants is not well understood, and the nutritional elements are unclear. Scientific evidence on the safety, detoxification, and nutrition of medicinal dietary plants of the Naxi people must be established before these medicinal dietary plants can be adopted by modern society to improve health and prevent diseases.

## Abbreviations

FL: Fidelity level
TCM: Traditional Chinese medicine
